# Attitudes Toward Transgender Men and Women: Development and Validation of a New Measure

**DOI:** 10.3389/fpsyg.2018.00387

**Published:** 2018-04-03

**Authors:** Thomas J Billard

**Affiliations:** Annenberg School for Communication and Journalism, University of Southern California, Los Angeles, CA, United States

**Keywords:** transphobia, transgender, attitudes, prejudice, psychometric scales

## Abstract

A series of three studies were conducted to generate, develop, and validate the Attitudes toward Transgender Men and Women (ATTMW) scale. In Study 1, 120 American adults responded to an open-ended questionnaire probing various dimensions of their perceptions of transgender individuals and identity. Qualitative thematic analysis generated 200 items based on their responses. In Study 2, 238 American adults completed a questionnaire consisting of the generated items. Exploratory factor analysis (EFA) revealed two non-identical 12-item subscales (ATTM and ATTW) of the full 24-item scale. In Study 3, 150 undergraduate students completed a survey containing the ATTMW and a number of validity-testing variables. Confirmatory factor analysis (CFA) verified the single-factor structures of the ATTM and ATTW subscales, and the convergent, discriminant, predictive, and concurrent validities of the ATTMW were also established. Together, our results demonstrate that the ATTMW is a reliable and valid measure of attitudes toward transgender individuals.

## Introduction

Transgender visibility in the United States has increased exponentially in recent years (Billard, 2016). Accordingly, researchers have dedicated increasing attention to the attitudes people hold toward transgender individuals and the factors underlying those attitudes (e.g., Tebbe and Moradi, 2012; Norton and Herek, 2013; Adams et al., 2016; Flores et al., 2017). However, the scales currently used to assess attitudes toward transgender individuals are insufficient in a number of ways (Morrison et al., 2017; Billard, 2018). It is therefore the purpose of this article to generate, develop, validate, and pilot a new scale to better, and more accurately, assess attitudes toward transgender men and women (ATTMW).

### Defining “transgender”

The term “transgender” refers to a broad range of social identities and gender presentations, and is used as such throughout the literature on attitudes toward transgender individuals (e.g., Hill and Willoughby, 2005; Nagoshi et al., 2008). “Transgender” is an umbrella term, under which fall people who live their daily lives as the gender opposite to that which is associated with the sex they were assigned at birth (*transgender men and women*), including those who seek medical intervention to align their bodies with the sex associated with their gender identity (*transsexual men and women*); people who identify outside of the binary categorization of gender (*non-binary*); people who cross-dress; drag performers; and (sometimes) intersex people—all people who cross (“trans-”) gender boundaries in some way. Because the term “transgender” encompasses so many identities, it is difficult to measure attitudes toward transgender individuals in the aggregate. Rather, attitudes must be measured such that transgender men and drag queens, for example, are not conflated, as attitudes toward the two will differ (Worthen, 2013).

Among other issues (Morrison et al., 2017; Billard, 2018), existing measures of attitudes toward transgender individuals fail on this front, only measuring attitudes toward transgender individuals on a broad level (Hill and Willoughby, 2005; Nagoshi et al., 2008; Walch et al., 2012; Kanamori et al., 2017). These scales are useful as measures of attitudes toward gender-nonconformity, but fail to distinguish between attitudes toward transgender men and women and gender-variant or transvestitic people. The scale presented in this article, however, more specifically addresses attitudes toward *transgender men* and *transgender women*.

### Existing transgender attitudes scales

Thus far, there are six published scales to measure attitudes toward transgender individuals: the Genderism and Transphobia Scale (GTS; Hill and Willoughby, 2005), Transphobia Scale (TS; Nagoshi et al., 2008), Attitudes Toward Transgendered Individuals Scale (ATTI; Walch et al., 2012), Transgender Attitudes and Beliefs Scale (TABS; Kanamori et al., 2017), Transsexual Prejudice Scale (Case and Stewart, 2013), and the Transprejudice Scale (for transgender women; Winter et al., 2009). However, only four of these will be the focus of our review—GTS, TS, ATTI, and TABS—because they have been used in studies other than the ones in which they were developed.

The most commonly used scale is Hill and Willoughby's (2005) GTS, which has been used in 29 studies at the time of writing. The scale was constructed to tap three theoretical domains identified by Hill (2002): (1) transphobia, or an emotional disgust at gender-nonconformity; (2) genderism, or an ideological orientation toward rigid gender division and sex-gender congruence; and (3) gender-bashing, or the physical, verbal, or psychological assault of gender-nonconforming individuals. However, their statistical analyses revealed two factors: Transphobia/Genderism (25 items) and Gender-Bashing (7 items). Yet while Hill and Willoughby (2005) demonstrated discriminant, convergent, predictive, and concurrent validity for the GTS—as have subsequent studies (e.g., Carrera-Fernández et al., 2014; Thomas et al., 2016)—the scale fails basic content validity checks (Billard, 2018) and exhibits psychometric inconsistencies.

Perhaps most significantly, as Nagoshi et al. (2008) noted, the GTS entirely fails to distinguish among the various identities under the umbrella of “transgender.” Moreover, the GTS does not refer to “transgender” individuals at all, referring instead to “masculine women,” “feminine men,” and men and women who exhibit alternately gendered characteristics. Additionally, the GTS includes behavioral items even though, as Walch et al. (2012) remarked, attitudinal scales are most effective when they do not include behavioral items as well. Regarding psychometric issues, Nagoshi et al. (2008) noted the high inter-correlations among the transphobia, genderism, and gender-bashing subscales, which indicates a lack of discriminant validity among the subscales. And in their validation of a short form of the GTS, Tebbe et al. (2014) further noted inconsistencies in factor structure across studies using the GTS, which suggests underlying conceptual problems with the factor structure identified by Hill and Willoughby (2005).

The TS and ATTI scales, however, have each been used in nine studies. Motivated by the shortcomings of the GTS, Nagoshi et al. (2008) developed the nine-item TS, which drew items directly from transgender activist Kate Bornstein's *My Gender Workbook*. These items loaded on one factor with a reliability of α = 0.82. However, the TS exhibits only limited validity. While Nagoshi et al. (2008) demonstrated discriminant and convergent validity and Weiner and Zinner (2015) demonstrated predictive validity for the TS, the scale fails basic content validity checks. For example, the scale items were merely pulled from Bornstein without any expert consultation, focus grouping, or reference against the extant literature. Moreover, the items generated for the scale refer broadly to issues of gender-nonconformity (e.g., “I avoid people on the street whose gender is unclear to me”) but not specifically on issues of transgender identity. Indeed, Nagoshi et al. (2008) definition of transphobia is “a person's degree of discomfort when encountering individuals who don't conform to conventional gender norms” (p. 523). Overall, it is an unproductive measure of attitudes toward certain identities under the transgender umbrella and cannot distinguish between prejudice against transgender men and women and non-binary individuals.

Walch et al.'s (2012) ATTI consists of 20 items derived primarily from existing homophobia scales, all of which loaded on one factor with a reliability of α = 0.95. While Walch et al. (2012) demonstrated discriminant and convergent validity and Riggs and Bartholomaeus (2015) demonstrated the predictive validity of the ATTI, Riggs and Bartholomaeus's (2016) application of the ATTI to an independent sample produced no statistically significant results. They hypothesized that may have been because the ATTI, “despite the amendments made, is relatively blunt” (Riggs and Bartholomaeus, 2016, p. 216). In addition to this major flaw, the ATTI fails basic content validity checks as the scale consistently uses the term “transgendered,” an incorrect term for referring to transgender individuals (Billard, 2016). Finally, the definition of “transgendered” Walch and colleagues developed to precede the scale explicitly includes “transsexuals and cross-dressers under the umbrella of transgender” (Walch et al., 2012, p. 1285), which, as previously noted, conflates attitudes toward individuals that must be measured as distinct. Thus, the ATTI is an insufficient measure of attitudes toward transgender individuals.

Most recently, Kanamori et al. (2017) developed the TABS scale, which has been used in two studies. The scale consists of 29 items, which loaded on three factors: interpersonal comfort (16 items), sex/gender beliefs (11 items), and human value (6 items). Thus far, the TABS has been demonstrated to have discriminant and convergent validity and a reliability of α = 0.98, but no predictive or concurrent validity (Kanamori et al., 2017). Moreover, the scale's content validity is questionable as the authors generated scale items in consultation with a Christian theology expert and deliberately oversampled Evangelical Christians in both the development and validation phases (Kanamori et al., 2017). In addition to being poorly justified, this approach renders the scale unrepresentative of general population attitudes and, despite the author's claims, fails to tap “religious nuances,” but, instead, taps right-wing Christian beliefs about transgender identity (Billard, 2018).

In sum, existing scales to measure attitudes toward transgender individuals fail on a number of fronts (see also Morrison et al., 2017). First, they fail to account for differences in attitudes toward transgender men and women, and, separately, non-binary transgender individuals (Worthen, 2013). Second, scale items reveal limited content validity as most scale items assess attitudes toward gender-nonconformity, rather than toward transgender individuals specifically (Billard, 2018). Third, these scales are not grounded in the attitudes actually held by the publics they intend to measure through using data assessing public attitudes toward transgender individuals. Instead they are generated from past measures of other prejudicial attitudes (e.g., homophobia) or from reviews of theoretical literature. Fourth, several of the existing scales measure only extreme levels of transphobia, but fail to assess the spectrum of ambivalent prejudice the public may hold. Fifth, existing scales often fail to measure general attitudes, instead using behavioral intentions or hypothetical behaviors as proxies for attitudes. Finally, each scale more accurately measures gender ideologies or attitudes toward the crossing of gender boundaries, but not attitudes toward transgender individuals. It is therefore the purpose of this article to offer a measure of attitudes toward transgender individuals that considers differences in attitudes toward transgender men and transgender women, and is grounded in the attitudes and beliefs expressed by members of the target public.

## Study 1: item generation and pilot testing

As a departure from previous scales, which have had limited basis in substantive data on public attitudes toward transgender people, a qualitative study was conducted to ensure the content validity of the new scale developed in Study 2. A sample of American adults responded to an open-ended questionnaire and their responses were coded qualitatively to generate scale items. A panel of survey construction and validation experts then piloted the generated items, which were refined according to their recommendations. This study thus ensures the items generated for the new scale accurately reflect the attitudes held by the American public and as such have sufficient content validity.

### Participants

Participants (*N* = 120) were recruited from Prolific, a human subjects crowdsourcing platform based at Oxford University Innovation. Prolific operates on a similar model to Amazon's Mechanical Turk (MTurk), but has been shown both to have more diverse, more naïve, more attentive, and less dishonest participants, and to produce higher quality data than MTurk (Peer et al., 2017). Prescreening was set such that only participants above 18 years of age living in the United States with task approval ratings above 90% were eligible.

To ensure a broad range of attitudes were captured—and because previous research has indicated gender and political affiliations as significant predictors of attitudes (e.g., Flores, 2015; Flores et al., 2017; Worthen et al., 2017)—the sample was stratified by sex (male/female) and partisan identification (Republican/Democrat). Thirty participants were recruited per strata for a total of 120 participants. Ages of participants ranged from 18 to 65 (*M* = 32.57, *SD* = 12.02). The majority of participants identified as heterosexual (75%) and White (71%). Large pluralities identified as Christian (47%) or non-religious (35%), and most held either an undergraduate degree (43%) or a secondary school diploma (32%).

### Procedure

Eligible participants were invited to participate through the Prolific dashboard and were offered modest financial compensation for their time. Participants completed an open-ended questionnaire probing their cognitive associations with the term “transgender,” lay definitions and etiologies of transgender identity, stereotypic perceptions of transgender people, personal feelings about transgender people, and political opinions about transgender rights (see Appendix A in Supplementary Material for full questionnaire). Average completion time for the questionnaire was 14 min.

Responses were analyzed qualitatively via thematic analysis (Braun and Clarke, 2006) to identify recurring attitudinal features and common perceptions of transgender individuals. This process entailed: (1) familiarization with the data by reading over participant responses, re-reading responses, and taking initial notes on recurring ideas; (2) systematically generating initial codes for noteworthy thematic features in the data; (3) condensing codes into a set of coherent themes; (4) re-coding data with the new thematic codes, producing a “map” of thematic variations; and (5) refining the details of each theme, applying relevant names, and definitions to each (see Braun and Clarke, 2006 for a full overview of the thematic analysis procedure). Drawing on this thematic analysis, a set of prospective scale items were generated to capture different variations on each identified theme. Most items contained close paraphrases or, where possible, direct quotations of participant responses. After generation, a panel of experts in survey construction and validation piloted tested the items, providing detailed feedback on item wording, comprehensibility, and fidelity to the construct of interest.

### Results

The qualitative thematic analysis following Braun and Clarke's (2006) guidelines informed the generation of 200 scale items as described above. Items were separated into 100 items regarding transgender men and 100 identical items regarding transgender women. Following pilot testing by a panel of experts, items were refined in accordance with their recommendations, resulting finally in the list of items presented in Appendix B in Supplementary Material.

### Closing remarks

The item generation and pilot testing methods employed in this study ensured the content validity of the scale developed in the following study. By drawing on qualitative data from a sample of American adults representing an equal distribution of men and women, as well as political affiliations, we can be confident that prospective scale items accurately reflect the attitudes toward transgender people held by the American public, thus making them suitable for a general measure of such attitudes in the American context. The use of thematic analysis to identify recurring attitudinal features and their variations further allowed for the generated items to capture core elements central to individual's attitudes toward transgender people while still providing sufficient diversity that the item reduction techniques used in the development of the final scale can identify the most powerfully predictive measures of American individual's attitudes from among the generated items.

## Study 2: scale development

The 200 items generated in Study 1 were administered to an independent sample of American adults and subjected to exploratory factor analysis (EFA). This analysis progressively eliminated items to reduce the number in the final scale, established the scale's factor structure, and assessed initial scale reliability. Items toward transgender men and transgender women were analyzed separately, developing two non-identical 12-item subscales (ATTM and ATTW) of the full scale (ATTMW).

### Participants

Participants (*N* = 293) were recruited from MTurk and screened so only United States residents above the age of 18 were eligible. Although MTurk occasionally suffers from data quality issues (Peer et al., 2017), scholars across several fields have convincingly demonstrated the reliability of results obtained from MTurk (e.g., Berinsky et al., 2012). Moreover, MTurk has been successfully used in the development and validation of other psychometric scales (e.g., Kanamori et al., 2017). However, to account for potential data quality issues, the present study limited participation to MTurk workers with task approval ratings above 95% and included numerous attention check questions in the questionnaire. Each participant received modest compensation for their time.

From the initial pool of 293 participants, 55 were eliminated for failing embedded attention checks or because of missing data, leaving a final sample of *N* = 238. Ages of participants ranged from 21 to 71 (*M* = 16.17, *SD* = 10.88). The majority (55%) of participants identified as male. Most participants identified as heterosexual (90%) and White (74%). The majority of participants identified as Christian (52%), with a large minority identifying as non-religious (41%). Most participants held either an undergraduate degree (48%) or a secondary school diploma (29%).

### Procedure

Participants completed a questionnaire consisting of demographic measures and the 200 items generated in Study 1. Each item took the form of a statement with which participants were asked rate their agreement on a 7-point Likert-type scale from 1 (*strongly disagree*) to 7 (*strongly agree*). Average completion time for the full study was 13 min.

The 200-item questionnaire was divided into two parts so participants rated their agreement with statements about transgender men first, then transgender women. Immediately preceding each half of the questionnaire, participants were provided with a definition of the term “transgender man” or “transgender woman,” respectively:

The term “transgender man” is used to describe people who were identified as female at the time of their birth but who currently live their daily lives as men.
The term “transgender woman” is used to describe people who were identified as male at the time of their birth but who currently live their daily lives as women.

Within each half of the questionnaire, items were rotated randomly to minimize potential order effects in participant's responses. Higher scores indicate greater anti-transgender prejudice, while lower scores indicate less prejudice.

### Results

Items regarding transgender men and transgender women were analyzed separately to develop two independent subscales. First, an EFA was run on the 100 items regarding transgender men using principal axis factoring with orthogonal (varimax) rotation. A high Kaiser-Meyer-Olkin value, KMO = 0.96, indicated high sampling adequacy and Bartlett's test of sphericity, $\chi_{\left(4,950\right)}^{2}$ = 28,161, *p* < 0.001, indicated sufficient inter-item correlations for analysis. Although there were 10 factors with eigenvalues >1, the first factor alone accounted for 53.9% of the overall variance, while the second factor accounted for only an additional 5.9%. Examination of the scree plot further indicated the appropriateness of a one-factor solution. To ensure the subscale consisted of items best representing the underlying construct, items with factor loadings below 0.7 were eliminated. This resulted in a final ATTM subscale consisting of 12 items. Factor loadings are presented in Table 1. The mean score was 3.5 (*SD* = 1.9) on a seven-point scale, indicating a relatively normal distribution. The subscale also displayed high reliability, α = 0.97, ω_*h*_ = 0.93^1^

**Table 1:** Factor loadings for EFA and CFA for the ATTM and ATTW subscales.

**Item**	**EFA loading**	**CFA loading**
**ATTM**		
Transgender men will never really be men	0.80	0.83
Transgender men are not really men	0.80	0.89
Transgender men are only able to look like men, but not be men	0.77	0.79
Transgender men are unable to accept who they really are	0.76	0.82
Transgender men are trying to be someone they're not	0.76	0.80
Transgender men seem absolutely normal to me. (*R*)^*^	0.74	0.58
Transgender men are denying their DNA	0.72	0.75
Transgender men cannot just “identify” as men	0.72	0.74
Transgender men are misguided^*^	0.72	0.83
Transgender men are unnatural	0.71	0.79
Transgender men don't really understand what it means to be a man	0.70	0.77
Transgender men are emotionally unstable^*^	0.70	0.58
**ATTW**		
Transgender women will never really be women	0.84	0.86
Transgender women are only able to look like women, but not be women	0.83	0.88
Transgender women are not really women	0.83	0.90
Transgender women are trying to be someone they're not	0.82	0.85
Transgender women are unnatural	0.81	0.86
Transgender women don't really understand what it means to be a woman	0.81	0.76
Transgender women cannot just “identify” as women	0.80	0.82
Transgender women are unable to accept who they really are	0.79	0.88
Transgender women only think they are women^*^	0.76	0.78
Transgender women are defying nature^*^	0.75	0.84
Transgender women are denying their DNA	0.75	0.83
There is something unique about being a woman that transgender women can never experience^*^	0.70	0.68

Items marked with an asterisk (

* *) are unique to that subscale*.

A second EFA was run on the 100 items regarding transgender women, again using principal axis factoring with orthogonal (varimax) rotation. A high Kaiser-Meyer-Olkin value, KMO = 0.97, and significant Bartlett's test of sphericity, $\chi_{\left(4,950\right)}^{2}$ = 30,644, *p* < 0.001, were achieved for these items as well. Although there were 10 factors with eigenvalues >1, the first factor alone accounted for 56.4% of the overall variance, while the second factor accounted for only an additional 6.0%. Examination of the scree plot further indicated the appropriateness of a one-factor solution. Again items with factor loadings below 0.7 were eliminated, resulting in a final ATTW subscale consisting of 12 items non-identical to those in the ATTM. Factor loadings are presented in Table 1. The mean score was 3.6 (*SD* = 2.0), indicating a relatively normal distribution. The subscale also displayed high reliability, α = 0.98, ω_*h*_ = 0.93. The reliability of the combined ATTMW was also high, α = 0.99, ω_*h*_ = 0.93.

### Closing remarks

The results of a pair of exploratory factor analyses revealed two non-identical single-factor subscales of 12-items each, which independently assess attitudes toward transgender men and transgender women. Average scores on each fell just below the midpoint of the scale, indicating the scale serves as a strong measure of a range of attitudes. Particularly considering the generally liberal skew of MTurk participants (Berinsky et al., 2012), we might expect a representative sample to produce mean scores closer to the scale's midpoint. Results of the analyses also suggest the subscales are highly reliable and internally consistent, both separately and together as a full 24-item scale. The newly developed ATTMW scale can thus be further validated through investigation of its relationship with other constructs.

## Study 3: scale validation

The newly developed ATTMW was administered to a sample of undergraduate students along with additional measures used to establish the validity of the scale. First, confirmatory factor analysis (CFA) verified the single-factor structures of both subscales of the ATTMW and ensured good model fit. Second, the convergent, discriminant, predictive, and concurrent validities of the scale and its subscales were established.

As reviewed previously, there are multiple existing measures of attitudes toward transgender individuals. While these measures are limited in several ways—most significantly in that they measure attitudes toward gender-nonconformity generally, rather than attitudes toward transgender individuals specifically—we should still expect a strong measure of ATTMW to be closely related to other measures of transgender attitudes. Two measures are of particular relevance to assessing this relationship. First, the GTS, as the most commonly used measure of transgender attitudes, is an important benchmark against which to compare a new measure of the construct. Second, the ATTI, as one of the next most common measures, and as a measure more specifically focused on attitudes toward individuals who identify as transgender, offers a strong point of comparison for the ATTMW. Therefore, we hypothesized the full ATTMW scale, as well as the ATTM and ATTW subscales independently, would correlate highly with the GTS and the ATTI, demonstrating they measure the same basic construct of attitudes toward transgender individuals (i.e., convergent validity)^2^.

Existing measures of transphobia have also been shown to relate to beliefs about gender roles. Indeed, prejudice against transgender individuals is rooted, in part, in the challenges transgender identity poses to the presumed separation and immutability of our genders assigned at birth (Norton and Herek, 2013). Thus, measures of attitudes toward transgender individuals should be related to measures of gender role beliefs (e.g., Tebbe and Moradi, 2012; Norton and Herek, 2013). Additionally, transgender identity in the United States is often considered in the context of “LGBT” identity more generally, which relates biases toward “sexual minorities” and those toward gender minorities among the general public. As such, measures of attitudes toward transgender individuals are often related to measures of homophobia (e.g., Tee and Hegarty, 2006; Adams et al., 2016). However, if a measure truly assesses attitudes toward transgender individuals specifically, it should be distinct from measures of these other constructs (Worthen, 2013). Therefore, we hypothesized the ATTMW, ATTM, and ATTW would each correlate significantly with measures of gender role beliefs and homophobia, though these correlations should be lower than those with other measures of transgender attitudes, demonstrating the new scale measures a construct distinct from gender roles beliefs and attitudes toward sexual minorities (i.e., discriminant validity).

Moreover, it is generally accepted that social attitudes toward minority groups are related to opinions about policy affecting those groups (e.g., Krysan, 2000). Past research has demonstrated this is true of attitudes toward transgender individuals as well (Flores, 2015; Flores et al., 2017; Miller et al., 2017). Therefore, we hypothesized higher scores on the ATTMW, ATTM, and ATTW (indicating *more* prejudice) would predict *less* support for pro-transgender policy, demonstrating the ATTMW can predict opinions it should reasonably be able to predict (i.e., predictive validity).

Furthermore, past research has demonstrated that political orientation influences attitudes toward transgender individuals, finding in particular that liberals generally hold more positive attitudes than conservatives (Nagoshi et al., 2008; Flores, 2015; Worthen et al., 2017). Therefore, we hypothesized individuals who identify their political orientation as more left-wing would report higher scores on the ATTMW and its subscales than those who identify as more right-wing, demonstrating the ATTMW can differentiate between groups that are theoretically distinguishable (i.e., concurrent validity)^3^.

Finally, one of the most consistent predictors of attitudes toward transgender individuals identified in the past literature is gender. Specifically, women consistently hold more favorable and less negative attitudes toward transgender individuals (Hill and Willoughby, 2005; Tee and Hegarty, 2006; Nagoshi et al., 2008; Walch et al., 2012; Norton and Herek, 2013; Carrera-Fernández et al., 2014; Flores et al., 2017; Miller et al., 2017; Worthen et al., 2017). Thus, we hypothesized that (non-transgender) men would report more prejudiced attitudes toward transgender people than women, demonstrating that the ATTMW can replicate findings produced by existing measures of attitudes toward transgender people.

### Participants

Participants (*N* = 152) were recruited from undergraduate courses at a large university in the western United States. Participants were offered extra course credit as compensation for their time. From the initial pool of 152 participants, two were eliminated because of missing data, leaving a final sample of *N* = 150. Ages of participants ranged from 18 to 27 (*M* = 20.25, *SD* = 1.45). The majority (76%) of participants identified as female. Most participants identified as heterosexual (95%) and large pluralities identified as either White (49%) or Asian (27%). Most participants identified either as Christian (39%) or as non-religious (35%). Participant's years of formal schooling ranged from 12 to 20 (*M* = 14.95, *SD* = 1.54).

### Measures

All measures took the form of seven-point Likert-type scales ranging from *strongly disagree* (1) to *strongly agree* (7), except where otherwise stated.

#### Demographics

In addition to standard demographic questions, participants were asked to identify how many days in the last month they had attended religious services as an indication of religiosity (*M* = 0.85, *SD* = 1.44). Participants were also asked to identify their political orientation on two unmarked bipolar sliding scales—from Democrat (0) to Republican (10) and from Liberal (0) to Conservative (10)—which by default rest in the center. These two scores were average to create a single measure of political orientation (*M* = 3.32, *SD* = 2.30).

#### ATTMW

Both subscales of the 24-item ATTMW scale were included. The ATTM subscale was preceded by the aforementioned definition of “transgender man,” while the ATTW subscale was preceded by the definition of “transgender woman.” In order to minimize any potential order effects in participant's responses, items within each subscale were rotated randomly.

#### ATTI

Walch et al.'s (2012) ATTI was included to assess the ATTMW's convergent validity. The 20 items were tested for reliability (α = 0.95, ω_*h*_ = 0.76) and averaged to create a single scale.

#### GTS

Hill and Willoughby's (2005) full GTS was included as a further test of convergent validity. The 32 items were tested for reliability (α = 0.95, ω_*h*_ = 0.63) and averaged to create a single scale.

#### Gender role beliefs scale

Kerr and Holden's (1996) Gender Role Beliefs Scale (GRBS) was included as a test of discriminant validity. The GRBS is a unidimensional measure of beliefs about the appropriateness of particular gendered behaviors. Sample items include “Women with children should not work outside the home if they don't have to financially,” and “The initiative in courtship should usually come from the man.” The 20 items were tested for reliability (α = 0.88, ω_*h*_ = 0.77) and averaged to create a single scale.

#### Attitudes toward lesbians and gay men scale

The five-item revised short form of the Attitudes Toward Lesbians and Gay Men scale (ATLG-R-S5; Herek and McLemore, 2011) was included as an additional test of discriminant validity. The ATLG-R-S5 consists of two five-item subscales—Attitudes Toward Lesbians and Attitudes Toward Gay Men—which are combined as one overall 10-item measure of homophobia. Use of this short version is recommended over the full ATLG scale (Herek and McLemore, 2011). Sample items include “I think male homosexuals are disgusting” and “Lesbians are sick.” The 10 items were tested for reliability (α = 0.87, ω_*h*_ = 0.77) and averaged to create a single scale.

#### Marlowe-crowne social desirability scale

A 10-item short form of the Marlowe-Crowne Social Desirability scale (Crowne and Marlowe, 1960) developed by Strahan and Gerbasi (1972; MCSD-S10) was included as a final test of discriminant validity, as well as to assess whether responses to the ATTMW were distorted by social desirability bias. The MCSD-S10 was chosen because it outperforms other forms of the scale (Fischer and Fick, 1993). Sample items include “I never resent being asked to return a favor” and “There have been occasions when I felt like smashing things.” Response options were *true* or *false*, with five items for which each were the socially desirable responses. Responses were summated to form a scale ranging from 0 (no socially desirable answers) to 10 (all socially desirable answers; *M* = 4.32, *SD* = 1.99).

#### Policy support

Support for a fictional pro-transgender bill was measured with three items assessing whether participants think the bill should pass or not, whether they would want their representative to vote for a similar bill or not, and whether they would vote for a candidate who supported a similar bill or not. This measure was included as an assessment of predictive validity. Response options for each item ranged from *definitely not* (1) to *definitely yes* (5). The three items were tested for reliability (α = 0.96, ω_*h*_ = 0.96) and averaged to create a single scale.

### Procedure

Participants completed a questionnaire consisting of demographic measures, the ATTMW, the ATTI, the GTS, the GRBS, the ATLG-R-S5, and the MCSD-S10. Participants then read a mock news article about a proposed bill in Ohio that would allow transgender individuals to use the restroom appropriate to their gender identity in all places of public accommodation. After reading the article, participants responded to the policy support items before receiving a debriefing identifying the article as fictional. Average completion time for the full study was 21 min.

### Results

The single-factor structures of the ATTM and the ATTW subscales were verified via CFA. First, a CFA was run on the ATTM using weighted least squares-mean estimation (Rhemtulla et al., 2012). The fit of the model was strong, $\chi_{\left(54\right)}^{2}$ = 110.80, *p* < 0.001, comparative fit index (CFI) = 0.996, Tucker-Lewis index (TLI) = 0.995, root mean square error of approximation (RMSEA) = 0.036, standardized root mean residual (SRMR) = 0.05. The ATTM also exhibited high reliability, α = 0.94, ω_*h*_ = 0.84. The mean score was 2.4 (*SD* = 1.2) on a seven-point scale, indicating a somewhat positively skewed distribution. However, considering characteristics of the sample (age, gender, political orientation, education, religiosity) known to predict more positive attitudes toward transgender individuals (e.g., Nagoshi et al., 2008; Norton and Herek, 2013), it is to be expected that mean prejudice levels among the sample would be lower than those among the public. A second CFA with weighted least squares-mean estimation was run on the ATTW. The fit of this model was also strong, $\chi_{\left(54\right)}^{2}$ = 184.57, *p* < 0.001, CFI = 0.995, TLI = 0.993, RMSEA = 0.043, SRMR = 0.05. The ATTW also exhibited high reliability, α = 0.96, ω_*h*_ = 0.85. The mean score was 2.4 (*SD* = 1.4), indicating an acceptably normal distribution given sample characteristics. The reliability of the combined ATTMW was also high, α = 0.98, ω_*h*_ = 0.87. Factor loadings for both subscales are presented in Table 1.

Table 2 presents the intercorrelations among the ATTMW and its subscales, construct validity test variables, and the MCSD-S10 (see Appendix C in Supplementary Material for the full table of intercorrelations among all research variables in study 3, Table S1). A hypothesized, the ATTMW, ATTM, and ATTW correlated highly with both the ATTI and the GTS (0.73 ≤ *r* ≤ 0.81), indicating the new scale indeed measures the same construct of attitudes toward transgender individuals. The ATTMW, ATTM, and ATTW also correlated significantly with the GRBS and ATLG-R-S5. However, they correlated much less strongly than with the other measures of attitudes toward transgender individuals (0.44 ≤ *r* ≤ 0.61), indicating they represent related but distinct constructs. Furthermore, the ATTMW and its subscales correlated with the GRBS and ATLG-R-S5 less strongly than did the GTS and ATTI, which suggests the ATTMW and its subscales offer greater distinction from related constructs than their competitor scales. Moreover, the ATTMW, ATTM, and ATTW had no significant correlation with the MCSD-S10, which both further confirms the discriminant validity of the scale and indicates responses were not significantly affected by social desirability bias.

**Table 2:** Intercorrelations among variables in study 3 (*N* = 150).

	**1**	**2**	**3**	**4**	**5**	**6**	**7**	**8**
ATTMW	–							
ATTM	0.98^***^	–						
ATTW	0.98^***^	0.92^***^	–					
ATTI	0.78^***^	0.80^***^	0.73^***^	–				
GTS	0.80^***^	0.81^***^	0.76^***^	0.83^***^	–			
GRBS	0.59^***^	0.61^***^	0.54^***^	0.63^***^	0.69^***^	–		
ATLG-R-S5	0.50^***^	0.54^***^	0.44^***^	0.67^***^	0.61^***^	0.60^***^	–	
MCSD-S10	0.01	0.02	−0.002	0.10	−0.01	0.05	0.12	–

*** *p < 0.001*.

Table 3 presents the results of three ordinary least squares (OLS) regression models run to assess the predictive validity of the ATTMW. In each model, policy support was the dependent variable and demographic variables were included as controls. In Model 1 the full ATTMW was included, while the ATTM and ATTW were included in Models 2 and 3, respectively. As hypothesized, the ATTMW, ATTM, and ATTW were each significant predictors of support for pro-transgender policy such that higher scores on each scale (i.e., greater prejudice) predicted lower policy support (−0.17 ≤ β ≤ −0.18).

**Table 3:** Summary of regression analysis for variables predicting support for pro-transgender policy (*N* = 150).

	**Model 1**			**Model 2**			**Model 3**		
**Variable**	**β**	***b* (*SE*)**	**95% CI**	**β**	***b* (*SE*)**	**95% CI**	**β**	***b* (*SE*)**	**95% CI**
ATTMW	−0.18^*^	−0.15 (0.07)	[−0.29, −0.01]	–	–	–	–	–	–
ATTM	–	–	–	−0.18^*^	–.16 (0.08)	[−0.31, −0.01]	–	–	–
ATTW	–	–	–	–	–	–	−0.17^*^	−0.13 (0.06)	[−0.25, −0.0001]
Age	−0.16^*^	−0.12 (0.06)	[−0.23, −0.01]	−0.16^*^	−0.11 (0.06)	[−0.23, −0.004]	−0.16^*^	−0.12 (0.06)	[−0.23, −0.01]
Gender (male)	−0.04	−0.10 (0.20)	[−0.50, 0.30]	−0.03	−0.08 (0.20)	[−0.48, 0.32]	−0.05	−0.12 (0.20)	[−0.51, 0.28]
Sexuality (hetero)	0.03	0.16 (0.40)	[−0.63, 0.95]	0.03	0.13 (0.40)	[−0.66, 0.92]	0.04	0.19 (0.40)	[−0.61, 0.98]
Race (white)	−0.10	−0.21 (0.18)	[−0.55, 0.14]	−0.10	−0.21 (0.18)	[−0.56, 0.14]	−0.10	−0.21 (0.18)	[−0.56, 0.14]
Religion (not Christian)	−0.02	−0.03 (0.17)	[−0.38, 0.31]	−0.01	−0.03 (0.17)	[−0.37, 0.31]	−0.02	−0.04 (0.17)	[−0.38, 0.31]
Religiosity	−0.08	−0.06 (0.06)	[−0.18, 0.06]	−0.08	−0.06 (0.06)	[−0.18, 0.06]	−0.09	−0.06 (0.06)	[−0.18, 0.05]
Political orientation	−0.29^**^	−0.13 (0.04)	[−0.22, −0.05]	−0.29^**^	−0.13 (0.04)	[−0.22, −0.05]	−0.30^**^	−0.14 (0.04)	[−0.22, −0.05]
*Adjusted R^2^*	0.15			0.15			0.15		
*F*	4.31^***^			4.31^***^			4.31^***^		

*CI, confidence interval*.

* *p ≤ 0.05*,

** *p < 0.01*,

*** *p < 0.001*.

A series of logistic regressions were run to determine whether the ATTMW could successfully be used to differentiate between groups that are theoretically distinguishable—in this case, those who are politically left-wing and those who are politically right-wing. First, political orientation was separated into quartiles. The top two quartiles (right-wing) and bottom two quartiles (left-wing) were then collapsed into binary groups. Results of the first logistic regression indicated the ATTMW was a significant predictor of political orientation, odds ratio (*OR*) = 1.83, 95% CI = [1.36, 2.47], *p* < 0.001, and the model was statistically significant, Nagelkerke's *R*^2^ = 0.16, $\chi_{\left(1\right)}^{2}$ = 18.86, *p* < 0.001. Results of the second model indicated the ATTM, too, was a significant predictor, *OR* = 1.97, 95% CI = [1.43, 2.71], *p* < 0.001, Nagelkerke's *R*^2^ = 0.18, $\chi_{\left(1\right)}^{2}$ = 21.04, *p* < 0.001. Results of the final model indicated the same for the ATTW, *OR* = 1.64, 95% CI = [1.26, 2.13], *p* < 0.001, Nagelkerke's *R*^2^ = 0.13, $\chi_{\left(1\right)}^{2}$ = 15.59, *p* < 0.001.

Finally, to examine potential differences in attitudes toward transgender people between (non-transgender) men and women we ran a series of independent samples *t*-tests. For all three tests (differences in ATTMW, ATTM, and ATTW scores) Levene's test for equality of variances was non-significant, indicating homogeneity of variance in the dependent variable across both groups (men and women). For the ATTMW, men (*M* = 2.91, *SD* = 1.39) reported significantly more prejudiced attitudes than women (*M* = 2.19, *SD* = 1.21), *t*_(148)_ = −2.98, *p* = 0.003, Cohen's *d* = 0.55. Men (*M* = 2.94, *SD* = 1.34) also reported significantly more prejudiced attitudes than women (*M* = 2.18, *SD* = 1.13) for both the ATTM, *t*_(148)_ = −3.32, *p* = 0.001, Cohen's *d* = 0.61, and the ATTW (*M*_men_ = 2.88, *SD*_men_ = 1.48; *M*_women_ = 2.21, *SD*_women_ = 1.35), *t*_(148)_ = −2.57, *p* = 0.01, Cohen's *d* = 0.47. Thus, as hypothesized, men held more prejudiced attitudes toward transgender people than women.

### Closing remarks

CFA confirmed the single-factor structures of the ATTM and ATTW and indicated good fits for both subscales. As expected, the ATTMW and its subscales were highly correlated with existing measures of attitudes toward transgender individuals, establishing the scale's convergent validity. Additionally, the ATTMW and its subscales correlated significantly with related measures of gender role beliefs and homophobia. However, while these correlations were quite large, they were notably smaller than the correlations with other measures of attitudes toward transgender individuals. This suggests the ATTMW is highly related to, yet still distinct from, these measures of gender and sexuality attitudes, thus establishing the scale's discriminant validity. The results of this study also demonstrated that, as expected, the ATTMW and its subscales significantly predicted support for pro-transgender policy, establishing the scale's predictive validity. Moreover, the concurrent validity of the ATTMW and its subscales was established by confirming the scale's ability to predict the hypothesized difference in attitudes between politically left-wing and right-wing participants. Finally, we demonstrated that the ATTMW can replicate the findings of studies that used alternate measures of attitudes toward transgender people by confirming differences in (non-transgender) men and women's attitudes. Taken together, the results of this study illustrate the newly developed ATTMW scale is a valid and useful measure of ATTMW, which outperforms existing measures of attitudes toward transgender individuals.

## Discussion

Across the three studies presented in this article, we generated and developed a new scale with which to independently assess attitudes toward transgender men and women and established the reliability and validity of the scale. The resultant ATTMW scale represents a significant contribution to the study of attitudes toward transgender people, as the scale outperforms existing measures of the same or similar constructs (see Morrison et al., 2017).

In the first study, we generated 200 prospective scale items based on a qualitative thematic analysis of 120 American adult's responses to an open-ended questionnaire probing their cognitive associations with the term “transgender,” lay definitions and etiologies of transgender identity, stereotypic perceptions of transgender people, personal feelings about transgender people, and political opinions about transgender rights. These 120 adults represented an even division both of (non-transgender) men and women and of right- and left-wing political affiliations. As such, the items reflected the breadth of public perceptions of transgender people in the United States, ensuring the content validity of the resulting scale. Items were further pilot tested by a panel of survey construction and validation experts to refine the item's content.

In the second study, we administered the 200 prospective scale items to an independent sample of 238 American adults. Although participants constituted a convenience sample, they were drawn from an online platform that has been found to be more representative of the general population than typical convenience samples (Berinsky et al., 2012). Responses were then subjected to principal axis factoring (a form of EFA), which revealed 2 non-identical 12-item subscales—ATTM and ATTW—of the full 24-item ATTMW. Initial tests of the scale's Cronbach's alpha(s) and hierarchical omega(s) indicated the scale is highly reliable (α = 0.97 − 0.99, ω_*h*_ = 0.93).

In the final study, we administered a survey consisting of the newly developed ATTMW and a series of validity-testing measures to a sample of 150 undergraduate students in the U.S. First, CFA confirmed the single-factor structures of both subscales. Then a series of correlation and regression analyses established the convergent, discriminant, concurrent, and predictive validities of both subscales independently and of the full scale combined. Further analyses confirmed the scale's continued reliability (α = 0.94 − 0.98, ω_*h*_ = 0.84 − 0.87).

Taken together, the results of all three studies demonstrate the total ATTMW scale, as well as its subscales independently, offers a reliable, valid, and useful measure of attitudes toward transgender men and women. Whereas no existing measures of attitudes toward transgender people have yet established all forms of validity and reliability (Morrison et al., 2017), we have come closer than our predecessors, having established every form of validity and reliability except for test-retest reliability, which was omitted from our studies due to concerns about collecting personally identifiable information in studies of such a sensitive nature. Our study further improves upon existing measures by (1) basing its items on the stated beliefs and attitudes of the general public, rather than merely on literature reviews or “expert” opinion; (2) separating out attitudes toward transgender men and toward transgender women, as attitudes toward different identities under the transgender umbrella must be considered as distinct, rather than uniform across identity categories (Worthen, 2013); and (3) performing better in statistical tests of discriminant validity, indicating that our scale is more distinct from associated but separate constructs than previous measures.

While the ATTMW is indeed a significant advancement on existing measures of attitudes toward transgender people, its limits must be noted. As discussed above, several distinct identities fall under the umbrella of “transgender,” and thus attitudes toward each must be measured separately. The current scale measures attitudes toward two identities under that umbrella—transgender men and transgender women—but cannot be used to investigated attitudes toward any others. As it measures people's discomfort with broad issues of gender-nonconformity, Nagoshi et al.'s (2008) TS may provide a productive measure of attitudes toward non-binary identities, but other transgender identities may necessitate the development of new scales. Moreover, the ATTMW was generated and developed in an American context and its items may not be applicable outside the U.S. without further validation. Thus, existing scales that have been validated cross-nationally should be employed until or unless the ATTMW is validated in additional cultural contexts.

In methodological terms, issues of sampling present a few limitations. Most significantly, due to limited resources, for no study was probability sampling employed. As such the results of each study are unlikely to be perfectly representative of the U.S. population's attitudes toward transgender men and women. Indeed, for both the MTurk and undergraduate student samples, demographic characteristics indicate that participants were more liberal than the U.S. population in general, and the latter sample is of course much younger than the general population. Moreover, data from MTurk workers sometimes suffers from quality issues (Peer et al., 2017), though precautions were taken in the present study to ensure the best quality data possible.

Future studies seeking to address these and other shortcomings should be undertaken. First, future studies should seek to establish the test-retest reliability of the ATTMW. Studies should also be undertaken to validate the ATTMW using nationally representative probability samples. Additionally, short forms of the scale should be generated for ease of use in longer research surveys. Following the example of the Attitudes Toward Lesbians and Gay Men short forms (Herek and McLemore, 2011), two variants could be created: a first that allows direct comparisons of attitudes toward transgender men and toward transgender women and a second that foregrounds those items which tap attitudinal elements distinct between the two identities. Finally, the ATTMW should be employed in future investigations of how attitudes toward transgender men and women differ and of the factors underlying those differences, as prior scales measuring attitudes toward transgender people have not enabled such analyses to be conducted.

## Ethics statement

This study was carried out in accordance with the recommendations of 45 CFR 46.101(b) category (2), University Park Institutional Review Board at the University of Southern California with written informed consent from all subjects. All subjects gave written informed consent in accordance with the Declaration of Helsinki. The protocol was approved by the University Park Institutional Review Board at the University of Southern California.

## Author contributions

The author confirms being the sole contributor of this work and approved it for publication.

### Conflict of interest statement

The author declares that the research was conducted in the absence of any commercial or financial relationships that could be construed as a potential conflict of interest.

## Footnotes

^1^In addition to Cronbach's alpha, reliabilities were assessed via hierarchical omega, which offers a more robust assessment of scale reliability and internal consistency (Peters, 2014).

^2^Also known as concurrent known-instruments validity (Abell et al., 2009).

^3^When convergent validity is referred to as concurrent known-instruments, this may be referred to as concurrent known-groups validity (Abell et al., 2009).
