# Supplementary material for: Attitudes Toward Transgender Men and Women: Development and Validation of a New Measure
Source: Front Psychol. 2018 Apr 3;9:387. doi: 10.3389/fpsyg.2018.00387 (PMC5891633; doi:10.3389/fpsyg.2018.00387)
Supplement: Supplementary file 1 [file Table1.docx]

**Appendix C – Correlations Among Research Variables for Study 3**

Table 1

*Correlation Among Research Variables for Study 3 (N = 150)*

| Variable | *M* (*SD*) | 1. | 2. | 3. | 4. | 5. | 6. | 7. | 8. | 9. | 10. | 11. | 12. | 13. |
| --- | --- | --- | --- | --- | --- | --- | --- | --- | --- | --- | --- | --- | --- | --- |
| 1. Age | 20.25 (1.45) | — |  |  |  |  |  |  |  |  |  |  |  |  |
| 1. Educ | 14.95 (1.55) | .65*** | — |  |  |  |  |  |  |  |  |  |  |  |
| 1. Relig | .85 (1.44) | -.10 | -.04 | — |  |  |  |  |  |  |  |  |  |  |
| 1. Politic | 3.32 (2.30) | -.11 | -.16 | .21* | — |  |  |  |  |  |  |  |  |  |
| 1. ATTMW | 2.37 (1.29) | .01 | -.06 | .14 | .39*** | — |  |  |  |  |  |  |  |  |
| 1. ATTM | 2.36 (1.22) | .01 | -.04 | .15 | .40*** | .98*** | — |  |  |  |  |  |  |  |
| 1. ATTW | 2.37 (1.41) | .01 | -.07 | .12 | .37*** | .98*** | .92*** | — |  |  |  |  |  |  |
| 1. ATTI | 2.23 (.94) | .09 | -.02 | .19* | .46*** | .78*** | .80*** | .73*** | — |  |  |  |  |  |
| 1. GTS | 2.00 (.82) | .03 | -.07 | .158 | .41*** | .80*** | .81*** | .76*** | .83*** | — |  |  |  |  |
| 1. GRBS | 2.77 (.79) | .05 | -.09 | .21** | .43*** | .59*** | .61*** | .54*** | .63*** | .69*** | — |  |  |  |
| 1. ATLG | 2.12 (.96) | .14 | -.04 | .21* | .35*** | .50*** | .54*** | .44*** | .67*** | .61*** | .60*** | — |  |  |
| 1. MCSD | 4.32 (1.99) | .13 | .06 | -.06 | -.01 | .01 | .02 | -.002 | .10 | -.01 | .05 | .12 | — |  |
| 1. Policy | 3.74 (1.05) | -.12 | .01 | -.16* | -.34*** | -.32*** | -.33*** | -.31*** | -.42*** | -.37*** | -.30*** | -.43*** | -.03 | — |
| *Note:* Educ = Education; Relig = Religiosity; Politic = Political Ideology; ATTMW = Attitudes Toward Transgender Men and Women; ATTM = Attitudes Toward Transgender Men; ATTW = Attitudes Toward Transgender Women; ATTI = Attitudes Toward Transgendered Individuals; GTS = Genderism and Transphobia Scale; GRBS = Gender Role Beliefs Scale; ATLG = Attitudes Toward Lesbians and Gay Men; MCDS = Marlowe-Crowne Social Desirability; Policy = Policy Support  **p* ≤ .05, ***p* < .01, ****p* < .001 | | | | | | | | | | | | | | |
