# Supplementary material for: Attitudes Toward Transgender Men and Women: Development and Validation of a New Measure
Source: Front Psychol. 2018 Apr 3;9:387. doi: 10.3389/fpsyg.2018.00387 (PMC5891633; doi:10.3389/fpsyg.2018.00387)
Supplement: Supplementary file 2 [file DataSheet1.docx]

**Appendix A – Open-ended Questionnaire for Study 1**

**Q1. What words come to mind when you think of the term “transgender”? List as many as you can.**

[OPEN RESPONSE]

**Q2. How would you define the term “transgender”?**

[OPEN RESPONSE]

**Q3. The term “transgender man” is used to describe people who were identified as female at the time of their birth but who currently live their daily lives as men.
What stereotypes do you think other people hold about transgender men?**

[OPEN RESPONSE]

**Q4. The term “transgender woman” is used to describe people who were identified as male at the time of their birth but who currently live their daily lives as women.
What stereotypes do you think other people hold about transgender women?**

[OPEN RESPONSE]

**Q5. If your friend’s teenager came out as transgender, how do you think they would react?**

[OPEN RESPONSE]

**Q6. Think about a transgender person you know of, either in real life or through the media. Does someone come to mind?**

000 NO 🡪 GO TO Q6A

001 YES 🡪 GO TO Q6B

**Q6A. The following transgender people have been in the media a lot. How familiar are you with each of them?**

**Laverne Cox**

**Chaz Bono**

**Caitlyn Jenner**

**Janet Mock**

**Laura Jane Grace**

**Jamie Clayton**

**Lana Wachowski**

**Chelsea Manning**

**Andrea Pejić**

001 NOT FAMILIAR AT ALL 🡪 GO TO Q7

002 SLIGHTLY FAMILIAR 🡪 GO TO Q7

003 MODERATELY FAMILIAR 🡪 GO TO Q6B

004 VERY FAMILIAR 🡪 GO TO Q6B

005 EXTREMELY FAMILIAR 🡪 GO TO Q6B

**Q6B. How would you describe them as a person?**

[OPEN RESPONSE]

**Q7. Transgender issues have been in the news a lot recently. List as many transgender issues as you can recall hearing about. If you can’t recall hearing about any issues, simply write, “None.”**

[OPEN RESPONSE] 🡪 IF “NONE,” GO TO Q7A; OTHERWISE, GO TO Q7B.

**Q7A. The following transgender issues have been in the news a lot recently. How much have you heard about each of them?**

**Transgender use of public restrooms**

**Transgender students’ use of school changing/locker rooms**

**Children transitioning genders**

**Transgender people being placed in gendered prisons**

**Companies refusing service to transgender people**

**Employers firing/refusing to hire transgender people**

**Transgender healthcare**

001 NONE AT ALL 🡪 GO TO CLOSE

002 A LITTLE 🡪 GO TO CLOSE

003 A MODERATE AMOUNT 🡪 GO TO Q7B

004 A LOT 🡪 GO TO Q7B

005 A GREAT DEAL 🡪 GO TO Q7B

**Q7B. Which of these issues do you think are most important? Why?**

[OPEN RESPONSE]

**Appendix B – Items Generated in Study 1**

**ATTM**

1. A transgender man’s gender history is no one else’s business.
2. Children should not be exposed to transgender men in the media.
3. Children should not be exposed to transgender men.
4. I could not stay friends with someone who came out as a transgender man.
5. I have a right to know if someone is a transgender man.
6. I would be devastated if someone I love came out as a transgender man.
7. I would be embarrassed to be seen with a transgender man.
8. I would be embarrassed to have a transgender man in my family.
9. I would be uncomfortable if a transgender man asked me out.
10. I would not be comfortable using a public restroom at the same time as a transgender man.
11. I would not feel comfortable being around a transgender man.
12. It takes a lot of courage for transgender men to be honest about who they are.
13. Someone who comes out as a transgender man is obviously very confused.
14. The issues facing transgender men are not important to society.
15. The issues facing transgender men are not important.
16. The issues facing transgender men are very important.
17. The lives of transgender men are valuable.
18. There is something unique about being a man that transgender men can never experience.
19. Transgender men are a danger to children.
20. Transgender men are a negative influence on children.
21. Transgender men are ashamed of being the sex they were born.
22. Transgender men are attention seeking.
23. Transgender men are brave.
24. Transgender men are deceitful.
25. Transgender men are defying nature.
26. Transgender men are denying their DNA.
27. Transgender men are disgusting.
28. Transgender men are emotionally unstable.
29. Transgender men are empowered.
30. Transgender men are good role models for children.
31. Transgender men are great role models for our society.
32. Transgender men are immoral.
33. Transgender men are inspirational.
34. Transgender men are just acting out for attention.
35. Transgender men are just butch women.
36. Transgender men are just going through a phase.
37. Transgender men are just like everyone else.
38. Transgender men are just like other men.
39. Transgender men are just playing dress up.
40. Transgender men are like this because of their parents.
41. Transgender men are like this because they didn’t have a strong mother.
42. Transgender men are lying to themselves.
43. Transgender men are mentally ill.
44. Transgender men are messed up.
45. Transgender men are misguided.
46. Transgender men are misunderstood.
47. Transgender men are not as tolerated as they should be.
48. Transgender men are not really men.
49. Transgender men are only able to look like men, but not be men.
50. Transgender men are only like this because it’s trendy.
51. Transgender men are overly sexual.
52. Transgender men are perverted.
53. Transgender men are potential sex criminals.
54. Transgender men are probably like this because they were abused.
55. Transgender men are repulsive.
56. Transgender men are scary.
57. Transgender men are selfish.
58. Transgender men are sexual predators.
59. Transgender men are special like everyone else.
60. Transgender men are trying to be someone they’re not.
61. Transgender men are trying to change our society.
62. Transgender men are unable to accept who they really are.
63. Transgender men are unnatural.
64. Transgender men cannot have healthy relationships.
65. Transgender men cannot just “identify” as men.
66. Transgender men create their own problems.
67. Transgender men deserve to be accepted by their families.
68. Transgender men deserve to be accepted.
69. Transgender men deserve to be beaten up.
70. Transgender men deserve to be happy.
71. Transgender men deserve to be mocked.
72. Transgender men do not bother anyone else.
73. Transgender men do not care about how their decisions affect their families and loved ones.
74. Transgender men don’t really understand what it means to be a man.
75. Transgender men gross me out.
76. Transgender men have a mental illness that needs to be treated.
77. Transgender men have a mental illness.
78. Transgender men have been brainwashed into being like this.
79. Transgender men have chosen to be this way.
80. Transgender men have no moral character.
81. Transgender men just couldn’t make it as a member of their own sex.
82. Transgender men make me uncomfortable.
83. Transgender men need more protection from hate crimes.
84. Transgender men only think they are men.
85. Transgender men only want the benefits of being male.
86. Transgender men seem absolutely normal to me.
87. Transgender men should be treated like men.
88. Transgender men should go through conversion therapy to stop being transgender.
89. Transgender men should just kill themselves.
90. Transgender men should not be hated for how they live their lives.
91. Transgender men should not be tolerated.
92. Transgender men should seek therapy.
93. Transgender men want special treatment.
94. Transgender men want to destroy traditional values.
95. Transgender men want to force their beliefs on other people.
96. Transgender men want to lead straight women astray.
97. Transgender men want to trick women into sleeping with them.
98. Transgender men were probably too close to their father.
99. Transgender men will never really be men.
100. Transgender men would be better off dead.

**ATTW**

1. A transgender woman’s gender history is no one else’s business.
2. Children should not be exposed to transgender women in the media.
3. Children should not be exposed to transgender women.
4. I could not stay friends with someone who came out as a transgender woman.
5. I have a right to know if someone is a transgender woman.
6. I would be devastated if someone I love came out as a transgender woman.
7. I would be embarrassed to be seen with a transgender woman.
8. I would be embarrassed to have a transgender woman in my family.
9. I would be uncomfortable if a transgender woman asked me out.
10. I would not be comfortable using a public restroom at the same time as a transgender woman.
11. I would not feel comfortable being around a transgender woman.
12. It takes a lot of courage for transgender women to be honest about who they are.
13. Someone who comes out as a transgender woman is obviously very confused.
14. The issues facing transgender women are not important to society.
15. The issues facing transgender women are not important.
16. The issues facing transgender women are very important.
17. The lives of transgender women are valuable.
18. There is something unique about being a woman that transgender women can never experience.
19. Transgender women are a danger to children.
20. Transgender women are a negative influence on children.
21. Transgender women are ashamed of being the sex they were born.
22. Transgender women are attention seeking.
23. Transgender women are brave.
24. Transgender women are deceitful.
25. Transgender women are defying nature.
26. Transgender women are denying their DNA.
27. Transgender women are disgusting.
28. Transgender women are emotionally unstable.
29. Transgender women are empowered.
30. Transgender women are good role models for children.
31. Transgender women are great role models for our society.
32. Transgender women are immoral.
33. Transgender women are inspirational.
34. Transgender women are just acting out for attention.
35. Transgender women are just effeminate men.
36. Transgender women are just going through a phase.
37. Transgender women are just like everyone else.
38. Transgender women are just like other women.
39. Transgender women are just playing dress up.
40. Transgender women are like this because of their parents.
41. Transgender women are like this because they didn’t have a strong father figure.
42. Transgender women are lying to themselves.
43. Transgender women are mentally ill.
44. Transgender women are messed up.
45. Transgender women are misguided.
46. Transgender women are misunderstood.
47. Transgender women are not as tolerated as they should be.
48. Transgender women are not really women.
49. Transgender women are only able to look like women, but not be women.
50. Transgender women are only like this because it’s trendy.
51. Transgender women are overly sexual.
52. Transgender women are perverted.
53. Transgender women are potential sex criminals.
54. Transgender women are probably like this because they were abused.
55. Transgender women are repulsive.
56. Transgender women are scary.
57. Transgender women are selfish.
58. Transgender women are sexual predators.
59. Transgender women are special like everyone else.
60. Transgender women are trying to be someone they’re not.
61. Transgender women are trying to change our society.
62. Transgender women are unable to accept who they really are.
63. Transgender women are unnatural.
64. Transgender women cannot have healthy relationships.
65. Transgender women cannot just “identify” as women.
66. Transgender women create their own problems.
67. Transgender women deserve to be accepted by their families.
68. Transgender women deserve to be accepted.
69. Transgender women deserve to be beaten up.
70. Transgender women deserve to be happy.
71. Transgender women deserve to be mocked.
72. Transgender women do not bother anyone else.
73. Transgender women do not care about how their decisions affect their families and loved ones.
74. Transgender women don’t really understand what it means to be a woman.
75. Transgender women gross me out.
76. Transgender women have a mental illness that needs to be treated.
77. Transgender women have a mental illness.
78. Transgender women have been brainwashed into being like this.
79. Transgender women have chosen to be this way.
80. Transgender women have no moral character.
81. Transgender women just couldn’t make it as a member of their own sex.
82. Transgender women make me uncomfortable.
83. Transgender women need more protection from hate crimes.
84. Transgender women only think they are women.
85. Transgender women only want the benefits of being female.
86. Transgender women seem absolutely normal to me.
87. Transgender women should be treated like women.
88. Transgender women should go through conversion therapy to stop being transgender.
89. Transgender women should just kill themselves.
90. Transgender women should not be hated for how they live their lives.
91. Transgender women should not be tolerated.
92. Transgender women should seek therapy.
93. Transgender women want special treatment.
94. Transgender women want to destroy traditional values.
95. Transgender women want to force their beliefs on other people.
96. Transgender women want to lead straight men astray.
97. Transgender women want to trick men into sleeping with them.
98. Transgender women were probably too close to their mother.
99. Transgender women will never really be women.
100. Transgender women would be better off dead.
